# Glucocorticoids in breast cancer: physiologic mechanisms and relationship to risk, survival, and treatment response

**DOI:** 10.3389/fendo.2026.1952560

**Published:** 2026-10-05

**Authors:** Robert T. Chatterton, Peter H. Gann

**Affiliations:** 1 Department of Obstetrics and Gynecology, Physiology and Pathology, Feinberg School of Medicine, Northwestern University, Chicago, IL, United States; 2 Department of Pathology, University of Illinois at Chicago, Chicago, IL, United States

**Keywords:** breast cancer, glucocorticoids, immune system, metabolism, receptors

## Abstract

Almost every cell of the body expresses glucocorticoid receptors (GR). Cell density, isoform distribution, and responsiveness vary greatly among tissues with highest density in cells of the immune system. Well-documented basic studies describe functions of GR-cortisol complex in immune cells; inhibition of inflammation, promotion of apoptosis, inhibition of proliferation, and inhibition of transport into tissues. Basic studies also describe metabolic actions; promotion of gluconeogenesis, amino acid mobilization, impairment of insulin secretion, inhibition of glucose uptake, and expansion of visceral fat depots. Cortisol secretion is stimulated by psychological stress, and by physiological responses to pain, infection, and hypoglycemia. Stimulatory inputs from the brain on CRH and AVP biosynthesis, deficiencies in the negative feedback of cortisol, and insufficient stimulation of β-arrestin-1 by cortisol may all act to increase secretion of ACTH. Peripherally, cortisol activity is preserved by 11βHSD type I and is decreased at specific sites by 11βHSD type 2. In metastatic breast cancer, the normal circadian rhythm of serum and salivary cortisol is often disrupted, with great hour-to-hour variation and elevated concentrations during the afternoon nadir. Cortisol may increase cancer risk by making glucose available through glycogenolysis and gluconeogenesis, by inhibiting insulin promotion of glucose uptake, and by inhibiting IGF-I promotion of leukocytopoiesis, increasing the synthesis of glutamine synthetase, inhibiting ICAM-I and VCAM-1 for leukocyte recruitment, and increasing the expression of immune checkpoint receptors PD-1 and Tim3, and increasing Treg cells. Cortisol promotes the polarization of macrophages toward the anti-inflammatory M2 phenotype and decreases the M1 phenotype. It also binds to membrane-bound corticosteroid-binding globulin, activating adenylyl cyclase, suppressing T cell proliferation. Overall, elevated cortisol secretion may promote growth of breast cancer by providing a substrate for growth and by suppression of host defenses. However, by antagonizing the estrogen response the liganded receptor may inhibit the growth of estrogen-dependent breast cancer. This review is relevant at this time because of the many interactions of glucocorticoids with potential treatments for the several subtypes of breast cancer. Glucocorticoid agonists may benefit therapies utilizing DNA damage response, retinoid therapy, and PARP1 therapy but may oppose therapies of ER-negative cancers and immune checkpoint blockade therapy.

## Highlights

Glucocorticoids are generally increased in stress and in many illnesses including breast cancer. Many factors are involved in this process. They have many impacts on the processes by which breast cancers proliferate. They act to provide carbohydrate substrates for cancer growth, they suppress host defense by several mechanisms, and they interact both genomically and non-genomically with other transcriptional factors to modify the tumor microenvironment. Well-documented basic studies describe the many mechanisms in which glucocorticoids act in the body. Inferences to clinical findings with their limitations are included when appropriate.

## 1 Introduction

Cortisol is known to affect many physiological processes. Immunosuppressive and metabolic activities have been well documented, but how all of the many activities interact to affect the growth of breast cancer has not been reviewed in recent years. This review is primarily a mechanistic and conceptual synthesis of glucocorticoid biology in breast cancer. Relevant clinical data are included when such information clearly relates to the principle being presented, although inferences from correlative clinical data should not be considered conclusive.

## 2 Biochemical activities of cortisol

Cortisol is the primary glucocorticoid in humans. Corticosterone has much less glucocorticoid activity and although it is the primary glucocorticoid in many species, it circulates as less than 2% of total glucocorticoids in man. Resting levels of cortisol are not different in men and women. The glucocorticoids are lipid-soluble steroids. Solubility of cortisol is 25 mM in ethanol; water solubility is less than 0.003 mM. It can diffuse across plasma membranes to bind to the glucocorticoid receptor without the need for a transport protein. Approximately 80 percent of cortisol in the circulation is bound to corticosteroid-binding globulin (CBG) and 15% to albumin. The remainder is free and capable of diffusing across the plasma membranes. CBG is a 50–60 kDa glycoprotein that binds cortisol at a 1:1 molar ratio with a Ka of 76 × 10^6^ L/mol. It also binds progesterone with a Ka of 24 × 10^6^ L/mol (1).

Glucocorticoid receptors (GRs) are located in the cell cytoplasm and belong to the steroid receptor superfamily (NR3C) of ligand-dependent transcription factors (2). GR (NR3C1) is expressed in two isoforms: GRα and GRβ. GRα binds glucocorticoids (GCs) and are able to activate transcription; GRβ does not bind to any ligand and fails to activate transcription but higher levels of GRβ lead to increased cortisol resistance. The Type 2 GRα is responsible for most of the glucocorticoid effects in many different cell types. Type 1 GRα receptors bind both GC and mineralocorticoids (MC)(receptor superfamily NR3C2) with similar affinity and are primarily involved in sodium and potassium transport activity. Binding of GCs to GRs results in the dissociation of heat shock proteins prior to translocation to the nucleus. Activation of GR target gene promoter regions occurs by the interaction of the zinc-finger domains of classic homodimeric GRα with specific GR-responsive elements (GREs) in the 5’ promoter region of a set of genes, thereby activating transcription (3).

GR can also activate transcriptional networks via protein–protein interactions such as those with AP-1 (Jun/Fos) and NF-κB, and can result in a positive or negative transcriptional outcome depending on the target gene and cellular context (3, 4). The promoter regions of several genes involved in inflammatory processes, including cytokines, chemokines, and adhesion molecules, contain specific DNA sequences to which pro-inflammatory transcription factors NF-κB and/or AP-1 can bind (5). GR-GC binding to both AP-1 and NF-κB sites results in trans-repression of the target gene. Such genes play a central role in regulating inflammatory responses, particularly through control of cytokine and chemokine gene expression (6).

GCs maintain energy homeostasis and, during stress conditions, they maintain elevated blood glucose levels to facilitate fight-or-flight responses (7–9) by promoting gluconeogenesis and glycogen storage in the liver and by inhibiting glucose uptake by skeletal muscle and adipose tissue. GCs also stimulate pancreatic alpha cells to release glucagon, thereby promoting hyperglycemia and insulin resistance. Consequently, chronically high GC levels promote the development of metabolic diseases. In addition, GCs induce reactive oxygen (ROS) and reactive nitrogen species (RNS) and resulting DNA damage. They also inhibit cyclooxygenase-2 and phospholipase A2, thus inhibiting inflammation by suppressing prostaglandin, leukotriene, and thromboxane formation (10).

In the CNS GCs influence a variety of functions, including learning and memory consolidation (11) as well as mood and psychiatric disorders (12). Depression has been associated with elevated circulating levels of cortisol as a result of reduced negative feedback of cortisol on ACTH and to mineralocorticoid receptor dysfunction (13).

Cortisol plays an important role in limiting the access of leukocytes to sites of infection or cancer activity (2). It inhibits TNF-α and IL-1β cytokine production; it reduces the effects of ICAM-1 on the transmigration of neutrophils and monocytes across the endothelium; and it reduces the effects of VCAM-1 on the migration of lymphocytes, monocytes, and eosinophils.

Cortisol enhances the barrier properties of epithelial tissues by increasing the expression of tight junction (TJ) proteins (14). The endothelial cells within the capillary network are sealed by tight junctions that restrict paracellular permeability (14). Shang et al. (15) concluded that the barrier forming proteins of the TJs (both claudin-3 and claudin-4) mediate interactions with other cells *in vivo*, restraining growth and metastatic potential by sustaining the expression of E-cadherin and limiting β-catenin signaling. However, claudins may also have signaling activities that promote cell proliferation. How cortisol-induced expression of TJ proteins is involved in cell proliferation, transformation, and metastasis is not well understood (16). Nevertheless, experimental evidence suggests that TJ proteins are important players in the initiation and progression of cancer. Nevertheless, the overall response to cortisol may depend on the circumstances or stages of tumor development.

The full spectrum of cortisol activities has been described in detail in reviews by Sapolsky et al. (17), Coutinho et al. (18) and earlier by these investigators (19). They have described permissive, suppressive, stimulatory, and preparative functions of cortisol and proposed that stress-induced increases in glucocorticoid levels protect not against the source of stress itself but rather against the body’s normal reactions to stress, preventing those reactions from overshooting and threatening homeostasis. They proposed a hormetic effect, such that at lower concentrations, cortisol may be permissive, i.e., allowing the stress response to proceed, and at higher concentrations, it becomes suppressive. They also suggest that some enzymes, such as glutamine synthetase, are rapidly induced by glucocorticoids and that detoxification mediators are released during stress-induced activation of primary defense mechanisms. These detoxification mediators can lead to tissue damage if left unchecked (17). Thus, cortisol may play a beneficial role by protecting against toxic responses to stress, but persistently high concentrations of cortisol may suppress beneficial responses to stress.

Glucocorticoids also have stimulatory and permissive functions. Patients with Addison’s disease are notorious for growth retardation and intolerance to infection (20), indicating an essential role of adrenocorticosteroid hormones in their stimulatory or permissive functions. Patients with primary adrenal insufficiency frequently experience fatigue, fever, infection, vomiting and diarrhea (21). Certainly, normal levels of glucocorticoids may play an important role in maintaining fluid balance, adequate glucose levels, and the release of serum immunoglobulins from the storehouse of immunoglobulins in lymphoid tissue (22).

## 3 Formation and secretion of adrenocorticosteroids

Steroid secretion from the adrenal cortex is promoted by a series of events beginning with internal or external stimuli that alter body homeostasis (23). Such stimuli increase the transcription and release of hypothalamic corticotropin hormone (CRH), urocortin 1 (UCN1), urocortin 2 (UCN2), and urocortin 3 (UCN3) from neurons originating from the paraventricular nucleus of the hypothalamus. These tropic factors bind in the anterior pituitary to CRH-R, a G-protein coupled receptor (GPCR), for the release of adrenocorticotropic hormone (ACTH). Negative feedback by the cortisol-occupied glucocorticoid receptor (GR) at this level suppresses the release of CRH and other tropic factors.

Stress increases several stimulatory factors, including epinephrine, norepinephrine, and glutamate, via inhibition by GABAergic neurons, all of which may contribute to modulation of CRH release from the hypothalamus. In addition to CRH, neurons of the paraventricular nucleus (PVN) contain arginine vasopressin, oxytocin, neurotensin, enkephalin, and cholecystokinin, each with separate or modifying activities. Stress is an important positive stimulus for CRH; however, factors, described below, that inhibit negative feedback of the hypothalamic-pituitary-adrenal (HPA) pathway are also important in increasing the activity of CRH.

Information on the pathway of cortisol formation in the adrenal cortex is abundantly available; however, some information on specific aspects is appropriate for this review. Cortisol is synthesized in the adrenal cortex from cholesterol which is stored in the form of cholesterol esters. ACTH stimulates steroid synthesis by binding to the melanocortin 2 receptor (MC2R), a G protein-coupled receptor located on the surface of adrenal cortex cells. This interaction activates adenylyl cyclase, leading to the production of cyclic adenosine monophosphate (cAMP). cAMP then activates protein kinase A (PKA), which phosphorylates specific transcription factors, increasing transcription of steroid acute regulatory protein (StAR) for transfer of cholesterol to the inner membrane of mitochondria, and ultimately stimulates the conversion of free cholesterol to pregnenolone by P450scc oxidoreductase. The rate of formation of further metabolites of pregnenolone is dependent on the rate of pregnenolone formation and the activity of specific cytochrome P450 enzymes and are not individually promoted by ACTH. Hydroxylations at the C21 carbon of the steroid by CYP21A2 and at C11 by CYP11B1 occur during the formation of both cortisol and corticosteroids. Hydroxylation of pregnenolone at carbon 17 (C17) by CYP17 is necessary for cortisol biosynthesis but not for the synthesis of the other major glucocorticoid, corticosterone. Cleavage of the side-chain of pregnenolone leads to the formation of the C-19 steroids, primarily dehydroepiandrosterone (DHEA) and androstenedione. DHEA is then largely converted to DHEA sulfate before release from the adrenal cortex. The ratio of DHEA to cortisol secretion from the adrenal cortex depends on the activity of the 17,20-lyase activity of CYP17A1.

In addition to modulation within the hypothalamic-pituitary system, sympathetic innervation of the adrenal gland via the splanchnic nerve increases the sensitivity of the adrenal gland to ACTH (24). Sympathetic activity originates from the brain from perceived stress, hypoglycemia, or other hemodynamic threat. Under non-stress conditions, when elevated steroid secretion is not required, splanchnic neural activity appears to be inhibitory, whereas during stress conditions, when elevated steroid secretion is necessary, neural activity is excitatory. In earlier studies, splanchnicectomy was shown to have an inhibitory effect on corticosterone secretion after a variety of stressors in several species but had little effect on secretion in non-stressed animals (25).

### 3.1 β-arrestin

Glucocorticoids redirect the signaling profile of GPCRs via alterations in β-arrestin gene expression, revealing a paradigm for cross-talk between nuclear and cell surface receptors and a mechanism by which glucocorticoids alter the clinical efficacy of GPCR-based drugs (30).

Elevated levels of cortisol increase the transcription of β-arrestin-1 and inhibit the transcription of β-arrestin-2 by liganded GR acting on GREs in intron-1 and intron-11, respectively (26).

β-Arrestins act to regulate cortisol secretion by suppressing propagation of the CRH signal. Both β-arrestin isoforms have a negative effect on activated G-protein coupled receptors (GPCRs), suppressing the progression of POMC transcription (27, 28), and the effect may be increased by as much as 30-fold following phosphorylation of the receptor by G-protein receptor kinases (29). In the process they shift the balance of CRH signaling from G protein-dependent coupling toward more β-arrestin-1–dependent signaling (activation of ERK).

In this way promotion of β-arrestin by cortisol may participate in the negative feedback of cortisol on ACTH secretion. On the other hand, insufficient stimulation of β-arrestin-1 by cortisol or alteration in the affinity of GPCR by reduction in G-protein-receptor kinase activity would increase CRH-1 activity at its receptor, increasing ACTH (**Figure 1**). More work needs to be done on the stimuli that alter transcription of the β-arrestins.

**Figure 1:**
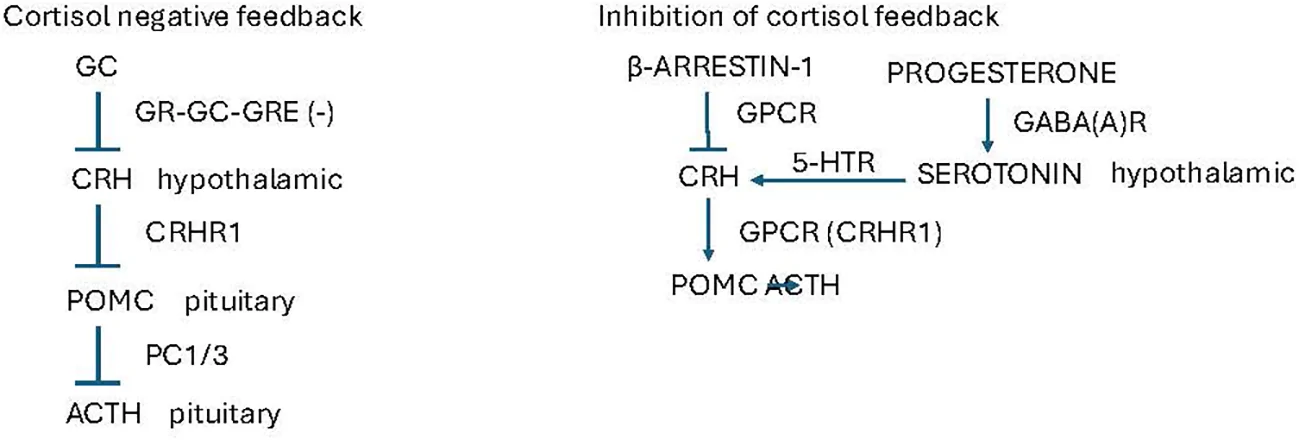
Hypothalamic control of ACTH secretion. GR-GC-GRE, liganded-bound GR binding to the GRE; the symbol (-) indicates inhibitory action. PC1 is the prohormone convertase; GABA(A)R, GABA(A) receptor.

### 3.2 Stimuli for activation of the hypothalamic-pituitary-adrenal axis

Acute social defeat stress in animal models triggers transient anxiety-like behaviors, accompanied by systemic inflammation and immediate-early gene (IEG) activation. Rapid activation of genes including c-fos, Egr-1, and cMyc occurs without prior protein synthesis. This results in elevated levels of pro-inflammatory cytokine Il-1β mRNA expression in the mouse brain and spinal cord, as well as increased CRH mRNA expression in the hypothalamus and elevated plasma corticosterone concentrations (30). The response is rapidly resolved. In contrast, chronic stress follows a dynamic progression. While early stress may be adaptively regulated by the body, prolonged or excessive stress can overwhelm the organism’s homeostatic capacity, leading to sustained overactivation of the HPA axis (31). Eventual downregulation of the adrenal response to ACTH may reduce the availability of cortisol, ultimately leading to elevated peripheral pro-inflammatory factors (e.g., TNF, IL-17, IL-1β, IL-6) and sustained neuroinflammation, marked by chronic microglial and astrocyte activation. This drives a state of chronic low-grade inflammation, which has been implicated in the development of various psychiatric disorders (32).

The reason for such decreases in cortisol responses over time is not well understood. Some authors have referred to “adrenal fatigue” while others see no evidence for a decrease in output of cortisol by the adrenal cortex in chronic stress (33).

In fact, major depression and other psychiatric conditions are often associated with hypercortisolism (13, 34). Conditions that chronically activate the hypothalamic–pituitary–adrenal axis (HPA) result in functional hypercortisolism and have been associated with cases of major depression, anorexia nervosa, bulimia nervosa, alcoholism, diabetes mellitus, simple obesity, polycystic ovary syndrome, obstructive sleep apnea syndrome, panic disorder, generalized anxiety disorder, shift work, and end-stage renal disease (35). Cessato et al. listed other high-risk conditions: arterial hypertension, osteoporosis and fractures, mood disorders, unusual infections and thrombotic events, and adrenal and pituitary incidentalomas (36). Whether the disease causes cortisol elevation or whether cortisol contributes to the disease is not clear for individual diseases. Surprisingly, an inflammatory condition, rheumatoid arthritis, is associated with slightly lower levels of cortisol (37) and significantly lower levels of androgens (38). Abraham et al. (39) found no association of obesity on urinary cortisol, and dexamethasone responses were not associated with BMI or weight. However, salivary cortisol was increased as a function of increasing BMI (P < 0.0001. This suggests that the increase is limited to the unbound fraction of cortisol in plasma. Erratic cortisol secretion in major depression may be a more characteristic feature of HPA axis dysregulation than hypercortisolism (40) as also shown in patients with metastatic breast cancer (41) and in some other conditions. It is clear that not only the concentration but the temporal pattern of secretion may be important in the response to cortisol (17).

Experimentally, severe physical and psychological stress during military survival training (42) and long distance running exercise (43) strongly stimulates cortisol elevation. The experience of free fall from the airplane (skydiving), which involves little physical exertion, also causes a large increase in serum and salivary cortisol (44).

Administered glucocorticoids differ from stress-induced cortisol secretion. Increased ACTH, whether due to stress or by administration, increases the secretion of all adrenocortical steroids. Cortisol formed in the fasciculata of the adrenal cortex acts to increase DHEA secretion in the reticularis zone by inhibiting 3β-hydroxysteroid dehydrogenase (45). Administration of prednisone or similar steroidal drugs provides glucocorticoid activity but suppresses ACTH and adrenal steroidogenesis. Therefore, the responses to ACTH and prednisone differ in that the glucocorticoid activity of the administered synthetic drugs is not opposed by DHEA and DHEA sulfate, nor is the activity of medullary chromaffin cells increased (46).

### 3.3 Extra-adrenal sources of cortisol

Thorough reviews of extra-adrenal steroidogenesis in several different tissues have been published by Taves et al. (33) and Gomez-Sanchez et al. (47). They have described studies demonstrating *de novo* biosynthesis of corticosteroids in primary lymphoid organs, the intestine, skin, brain, and adipose tissue; however, the precursor for the synthesis of these steroids has not been established. Evidence for local synthesis includes the detection of steroidogenic enzymes and high local corticosteroid levels after adrenalectomy. Numerous studies have shown the presence of CYP11A and mRNAs of other enzymes necessary for biosynthesis of pregnenolone and corticosterone in lymphoid tissues and other sites, and the extra-adrenal stimulation of corticosterone by CRH (48) and ACTH (49). Interestingly, 21-hydroxylase in the human brain and other extra-adrenal tissues differs from that in adrenal CYP21 (50, 51). It is present as a CYP2D6 isoform and is also abundantly expressed in lymphoid tissues (51).

The presence of local biosynthesis of corticosterone or aldosterone in adipose tissue (52), keratinocytes (53) or monocyte-macrophage lineage cells (54) may be relevant to growth of solid tumors in that, as membrane-permeable steroids, adrenocortical steroids produced in the surrounding adipose tissue may reduce the ability of tumor-infiltrating lymphocytes (TILs) to act on cells expressing tumor antigens. Although the breast is not specifically mentioned in these articles, steroidogenesis in surrounding tissues may be a source of glucocorticoids.

Acharya, et al. (54) reported that GR activation in CD8 cells was associated with reduced effector function in animal models for colon cancer and in human colorectal cancer. Local production by monocyte-macrophage cells was identified as the source of glucocorticoids in the tumor environment, and inhibition of steroidogenesis in those cells restored effector function in tumor infiltrating CD8 cells. Consistent with this, GC signaling was observed to be associated with resistance to checkpoint inhibitor therapy in preclinical models and human melanoma. The relevance of these findings for breast cancer represents an important topic for future research.

### 3.4 Role of the 11β-hydroxysteroid dehydrogenases

After secretion, oxidation of cortisol by 11β-hydroxysteroid dehydrogenase (HSD11β) type 2 may occur. This produces cortisone, the inactive form of the glucocorticoid. The inactivation of cortisol is important for reducing glucocorticoid activity in organs such as the kidney, where mineralocorticoid activity may be bolstered by high concentrations of circulating cortisol. HSD11β type 2 also protects adult lungs from the anti-proliferative effects of cortisol (55) and protects the brain from the mineralocorticoid activity of cortisol (56).

Alternatively, cortisone may be reduced to cortisol by HSD11β type 1, which can increase glucocorticoid activity in the liver, muscle, and adipose tissue where it serves metabolic functions. HSD11β type 1 can be induced by TNFα (57), inflammatory cytokines, and glucocorticoids in skin fibroblasts, synovial fibroblasts, and osteoblasts (57). Cytokines also induce HSD11β type 1 in macrophages (58, 59), T- and B-cells, and dendritic cells (60, 61), thus maintaining the active form of cortisol for its suppressive effects on the immune system. Thus, HSD11β type 1 aids in maintaining the active, reduced form of the steroid, while inflammatory cytokines are prevalent. The production of intra-tumoral cortisol may be increased within cancer cells of cancer types that express high levels of HSD11b1 or that downregulate HSD11b2. This is likely coming from tumor cells in addition to local adipose tissue or both according to Nakayama et al. (204). For example, TNBC with high expression of HSD11b1 have poor outcomes.

## 4 Modification of cortisol effects and availability

Circulating glucocorticoids are the primary negative feedback regulators of ACTH release. Glucocorticoids decrease the hypothalamic levels of CRH (62) by reducing CRH mRNA (63). Glucocorticoids also act at the pituitary level by inhibiting the effect of CRH on POMC transcription and ACTH release (64) and they increase the formation of β-arrestin as described above. Cortisol negative feedback of ACTH secretion involves GR acting on negative GREs in the promoter region of POMC. Inhibition at this level suppresses not only the effect of CRH on ACTH secretion but suppresses other agonists such as arginine vasopressin (AVP) and the CRH-related peptides urocortin 1 (UCN1), urocortin 2 (UCN2), and urocortin 3 (UCN3).

In women with metastatic breast cancer, the afternoon/evening suppression of cortisol levels that normally occurs is frequently reduced. As mentioned above, it is possible that GR availability to cortisol may be reduced by the binding of other steroids, or the interaction of the GR with the GRE in the promoter regions of the POMC and CRH genes may be reduced. It is also possible that the negative feedback of CRH on the CRH-R is deficient (62).

To evaluate the feedback regulation of cortisol secretion, dexamethasone, a 1.0 mg dose was administered to metastatic breast cancer patients at 2200 h (65). Salivary cortisol concentrations measured during the following day were found to be significantly suppressed in patients that had shown typically decreasing concentrations of salivary cortisol with time of day (P < 0.01), but salivary cortisol in patients with flattened salivary cortisol patterns was not suppressed, indicating the probability of failure of normal hypothalamic negative feedback in patients with flatter slopes.

Ceulemans et al. (66) also found that surgical patients with a lack of plasma cortisol suppression in the dexamethasone suppression test (DST) were associated with increased acute anxiety as determined by the State-Trait Inventory on the State scale (The Trait scale was not different) as well as elevated plasma cortisol levels. Depression assessed using the Hamilton scale was not significant. Other observations have also suggested that physiological or psychiatric stress may inhibit DST (67, 68). For example, in an experimental model employing rhesus monkeys, resistance to DST was associated with mean arousal ratings after nasogastric tube insertion (69). Although the DST is a proven method for determining whether excessive levels of cortisol are caused by a pituitary tumor, the reason for the reduction in negative feedback associated with metastatic breast cancer or major depression is not known.

Many aspects of feedback regulation complicate the investigation of this observation. The increase in cortisol levels in women with flattened profiles may be due to factors outside the hypothalamic-pituitary axis (HPA). Spiegel et al. (70) carried out a CRH activation test in 93 metastatic breast cancer patients. ACTH and cortisol levels in serum measured at intervals from -16 to 120 min were highly correlated (r=0.66, p<.0001, N = 74), indicating that the cortisol response of the adrenal gland could be attributed to CRH acting on ACTH, and no other stimuli were required.

### 4.1 Role of ultradian and diurnal rhythms of cortisol secretion

The extremely rapid pulsatile pattern exhibited in patients with flat cortisol patterns may alter the expression of several genes. According to Lightman and Conway-Campbell (71) dynamics of glucocorticoids interacting with their receptors represents a dynamic system that results in equilibrium throughout the neuroendocrine system. The dynamic state of cortisol levels is influenced by feed-forward and feedback loops. For example, non-ACTH inputs to the adrenals, such as the splanchnic nerve, and intra-adrenal clock mechanisms could modulate the pattern of this pituitary–adrenal oscillation by altering adrenal sensitivity to ACTH. The authors proposed that this continuous oscillatory activity is crucial for the optimal responsiveness of glucocorticoid-sensitive neural processes.

Ultradian glucocorticoid rhythms are highly conserved across mammalian species; however, their functional significance is not fully understood. Flynn et al. (72) demonstrated that pulsatile corticosterone replacement in adrenalectomized rats induced a pattern of GR binding at approximately 3,000 genomic sites in the liver at the pulse peak that was subsequently not found during the pulse nadir. In contrast, constant corticosterone replacement induced prolonged binding at most sites. In addition, each pattern induced markedly different transcriptional responses. In contrast to frequent pulses of secretion, constant corticosterone exposure induced significant effects on RNA polymerase II occupancy at the majority of gene targets, thus acting as a continuous regulatory signal for both the transactivation and repression of glucocorticoid target genes. Mitre-Aguilar et al. have presented a review of the effects of glucocorticoids on the circadian rhythm and the association of dysregulated clock genes and cancer (73). This is an additional mechanism by which glucocorticoids may be involved in promotion of breast cancer, but the significance of this effect is not known (74). Nevertheless, the importance of circadian and ultradian patterns which alter glucocorticoid responses cannot be overemphasized and may be the primary HPA defect in women with metastatic breast cancer.

### 4.2 Role of obesity in glucocorticoid-associated breast cancer progression

Obesity may be one of the factors that may result in elevated cortisol levels in patients with metastatic breast cancer. Obesity has been shown to be a risk factor for the development of hormone receptor-positive breast cancer in postmenopausal women but not in premenopausal women (75). The lack of association of obesity with breast cancer in premenopausal women has been attributed in part to the opposing beneficial effect of reduced exposure to estrogen as a result of oligomenorrhea in obese premenopausal women.

In humans, insulin resistance, associated with elevated cortisol concentrations, leads to obesity. Subclinical inflammation in visceral and subcutaneous adipose tissue of obese patients is commonly characterized by necrotic adipocytes surrounded by macrophages that form crown-like structures (CLS) (76). Such structures have been found in breast tissue of nearly 50% of obese patients at surgery. The severity of breast inflammation, defined as the CLS-B index, was correlated with the body mass index (P < 0.001) and adipocyte size (P = 0.01). NF-κB binding activity and elevated aromatase expression and activity were observed in the inflamed breast tissue of these women (76). Cortisol inhibits NF-κB at the transcriptional level (10) and may inhibit the function of M1 macrophages (those characterized by high levels of proinflammatory cytokines) in this situation.

### 4.3 Associated findings in other systems

Changes in the circadian rhythm of cortisol concentrations in patients with metastatic breast cancer appear to be similar to those in patients with mild autonomous cortisol secretion (MACS), with elevated afternoon/evening serum or salivary cortisol concentrations. MACS patients often have adrenocortical adenomas and abnormal urine steroid profiles but differ from metastatic breast cancer patients in that they have high glucocorticoid to androgen ratios and generally have higher nocturnal cortisol production (77). MACS patients had only 38% as much DHEA in 24-h urine as controls (77) while metastatic breast cancer patients had significantly elevated DHEA serum levels (78). Decreased circulating androgen in patients with MACS is contrary to the general stress response (17), which may be explained by the fact that the source of glucocorticoids is often an adrenocortical adenoma. Further studies should be conducted in this area. Aberrant cortisol circadian rhythms have also been associated with other circadian abnormalities (79), making it possible that these results reflect circadian abnormalities extending beyond the adrenocortical axis. Aberrant cortisol rhythms may be associated with hypersensitivity to stress (80) or may be associated with disruption of other endocrine and immune rhythms (81). Although the disrupted patterns of serum cortisol in shift workers are more extreme than those associated with metastatic breast cancer, the associations with reduced immune tolerance (82), impaired glucose metabolism, and increased inflammation (83, 84) may apply to some degree. Effects of abnormal hormonal patterns in combination with the psychological stress of cancer, may cause deterioration of disease resistance capabilities (85).

### 4.4 Paradoxical effects of stress on cortisol levels

Van der Pompe et al. (86) measured the serum ACTH and cortisol levels in women with early breast cancer, metastatic breast cancer, and controls. Prior to a stress test, serum cortisol levels were elevated two-fold in women with metastatic breast cancer and somewhat less in women with early-stage breast cancer, but ACTH levels were not different from those in controls. A stress test in which the participants recited a self-composed story resulted in a 23% decrease in serum cortisol in women with metastatic breast cancer and a 12% decline in women with early-stage breast cancer after 37 min. Although public recitation is considered to be stressful, the decrease in plasma cortisol that was observed brought levels back toward normal. Participation in the stress test may actually have felt supportive as part of a group activity leading to a lower stress response. The authors suggested that the decrease in serum cortisol may be due to increased metabolic clearance or it may be sequestered in sites outside the circulation. These observations on stress in women with metastatic breast cancer should be replicated in future studies. The observations of both cortisol elevation and suppression without concomitant changes in ACTH levels indicate that CRH and ACTH are not the only important factors regulating plasma cortisol levels.

Other studies have confirmed that some types of stress may actually be associated with decreased serum cortisol levels. For example, lower plasma cortisol levels were observed in male medical students preparing for a skydiving event compared to a similar control group of students, and there was a progressive decline in cortisol in blood samples taken from an indwelling catheter at 15-min intervals from the students traveling to the skydiving site (44). Another sample was taken immediately before entering the airplane for the skydive (**Figure 2**), shown as the arrow on the graph. Four additional samples were taken at 15-min intervals beginning when the volunteers touched the ground after the skydive. Even when entering the airplane, cortisol levels were not elevated. Only the actual experience of skydiving caused a rapid and profound increase in plasma cortisol levels. Similarly, a situation has been described in which soldiers waiting for an enemy attack had lower than normal glucocorticoid production, whereas the responsible officers had high levels (87). In addition, radio officers in planes involved in practice landings on aircraft carriers did not have elevated urinary 17-hydroxycorticosteroids despite the fact that they were in as much at risk as the pilots who had high urinary corticoid levels (88). It may be that people who have the ability to alter their situation produce a cortisol response and those who cannot do not produce a cortisol response. Repeated exposure without the ability to adapt may also cause a failure of the cortisol response. Animal studies may be relevant in this regard. In rats with evidence of a hyperactive pituitary-adrenal system, repeated exposure to stress results in decreased corticosterone levels (89). Constant high levels of CRH, which may be present during chronic stress, may cause downregulation of the CRH receptor in the pituitary (89).

**Figure 2:**
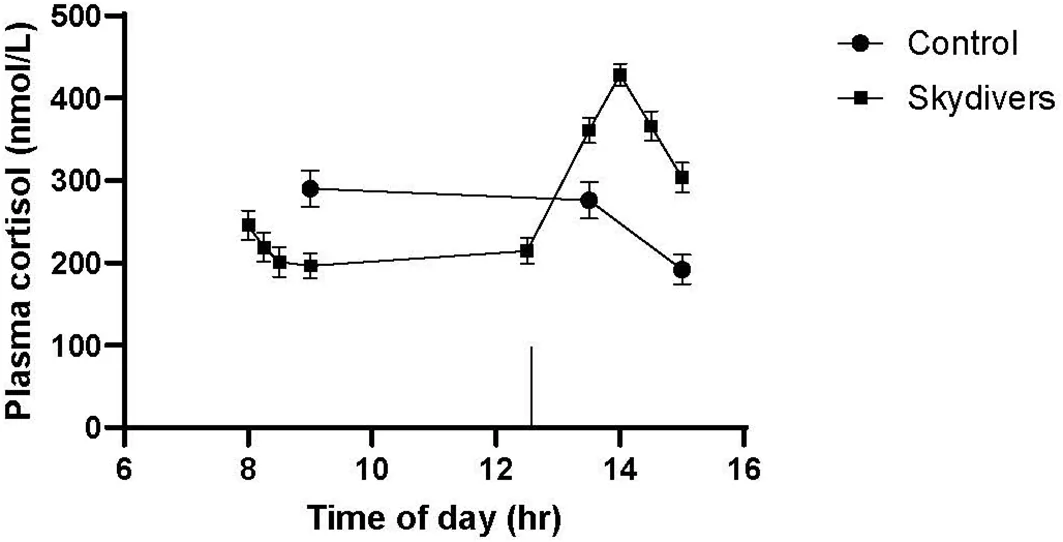
Plasma cortisol was measured in blood samples obtained from an indwelling catheter throughout the day of the skydive (solid circles) at the times of day indicated on the abscissa from 26 subjects. Samples from 15 control subjects were taken from a similar group of subjects who were not recruited for skydiving (open circles). The time of entering the airplane is indicated by the arrow on the graph. Standard errors of the means are shown. Adapted from Chatterton et al., 1997 (44).

Stress may result in elevated concentrations of serum free cortisol by interference with cortisol negative feedback at hypothalamic or pituitary levels. However, some forms of stress may act through mechanisms outside the HPA axis to suppress serum levels of cortisol. Although serum cortisol and ACTH are highly correlated in most physiological conditions, changes in CBG binding of glucocorticoids or metabolism may cause this relationship to fail.

## 5 Receptor interactions affecting cortisol actions

### 5.1 Dehydroepiandrosterone

Although adrenal cells that produce DHEA and cortisol reside mainly in different parts of the adrenal cortex, there is no evidence that a separate DHEA-stimulating factor exists. Adrenal DHEA biosynthesis is dependent upon ACTH stimulation. Nevertheless, the secretion of cortisol and DHEA at times diverge as a function of steroid 17,20-lyase activity (90). This is apparent with age, when the serum concentrations of DHEA increase more rapidly than cortisol in the first 30 years of life and then decline progressively after the age of 30 (91, 92) while cortisol remains relatively constant DHEA plays a significant role as an important modifier of the actions of cortisol (93). Cortisol is anti-inflammatory and immunosuppressive whereas DHEA activates the immune system (26, 94). The increased ratio of cortisol to DHEA with advanced age increases the exposure to the chronic effects of cortisol in the elderly and may be part of the reason that the incidence of cancer increases with age.

Higher serum DHEA concentration has been considered a potential risk factor for breast cancer. Odds ratios as high as four have been associated with elevated serum DHEA levels and breast cancer incidence in epidemiological studies. However, numerous experimental studies have shown that DHEA and DHEA sulfate reduce the risk of mammary cancer (reviewed by Chatterton in 2022 (95) and 2024 (78)) and DHEA may antagonize a number of cortisol responses. The mechanisms by which DHEA suppresses tumor growth includes the non-competitive inhibition of glucose-6-phosphate dehydrogenase, inhibition of cholesterol biosynthesis, immune suppression in virally induced breast cancer, enhancement of natural killer cell cytotoxicity by both DHEA and DHEAS, suppression of IL-6, and promotion of estrogen receptor beta expression (78).

Many of the diseases which have been designated as ‘cortisol-potentiated’ occur most frequently in the elderly and are generally associated with age-related conditions, e.g., type II diabetes, obesity, osteoporosis, endogenous depression, cardiovascular disease (93). Serum cortisol concentrations are generally maintained with age (96) while DHEA and DHEA sulfate, which counteract many functions of cortisol, decline with age. Thus, there is a loss of immune-activating DHEA with a relative abundance of immunosuppressive effects of cortisol. In a study by Parker et al. (97) basal serum cortisol levels were markedly increased in severe illness, whereas levels of the adrenal androgens DHEA and DHEAS tended to be lower (P < 0.01). The DHEA to cortisol and DHEAS to cortisol ratios were 4.4 and 3.4 times lower, respectively, in an ill group of patients in the ICU of a veteran’s hospital compared with those in the normal group (P < 0.001). Acute stimulation with adrenocorticotropic hormone (ACTH) demonstrated that the increase in cortisol was the same in normal and severely ill patients. However, the acute increase in serum DHEA concentration was significantly lower (P <0.05) in severely ill patients, indicating a decreased ACTH-stimulable DHEA reserve. Daily basal urinary unconjugated DHEA excretion was decreased, while cortisol excretion was increased, thereby decreasing the DHEA-to-cortisol ratio in the ill group by 85.4% (P < 0.001) relative to that in the normal group.

Therefore, we have postulated that the increased secretion of DHEA in breast cancer patients is a result of ACTH stimulation of adrenal steroidogenesis, which increases both DHEA and cortisol, although whether this applies to both ER-positive and TNBC has not been determined. The cortisol component suppresses immune activity and limits tumor access of immune cells while DHEA stimulates immune activity, opposing to some degree the effects of cortisol.

### 5.2 Progesterone

Progesterone is known to compete for the glucocorticoid receptor but inhibition varies by cell type. Kontula et al. (98) found that the relative binding affinity of progesterone for the GR in cultured lymphoma cells was 25% that of dexamethasone. A hundred-fold excess of progesterone inhibited induction of tyrosine aminotransferase in HTC cells by only 44%, yet a ten-fold excess of progesterone inhibited binding of dexamethasone in these cells by 70% (99). Thus, the effect was disproportionately less than would be predicted by binding affinity of progesterone. Potentially progesterone or its metabolite allopregnanolone may inhibit the activity of cortisol in its role in feedback inhibition of ACTH secretion but at normal plasma concentrations this effect would appear to be minimal. Nevertheless, during pregnancy, when plasma progesterone concentrations are elevated, serum cortisol levels (both CBG-bound and free) are increased. This has been observed in both rats (100) and humans (101) suggesting that progesterone interferes with cortisol negative feedback on ACTH secretion.

In addition, progesterone may have stimulatory effects in the brain on the stress release of CRH. In the ewe chronic progesterone administration increased the response to a hypotensive stressor (102). Plasma progesterone concentrations were exogenously increased to a mean of 7.6 ng/ml by subcutaneous implants. This was a significant increase over the 2.3 ng/ml in untreated ewes. The treatment had no effect on plasma cortisol or glucose concentrations. However, the ACTH response to a stressor (nitroprusside induced hypotension) was approximately 80% greater in the progesterone treated ewes. Because AVP was also increased in the treated ewes, it appears that the effect was at a hypothalamic or higher level. This is consistent with effects of progesterone and its metabolite allopregnanolone as modulators of GABA(A) receptors in the brain (103).

Progesterone acting via its metabolite allopregnanolone also increases serotonin turnover in the brain (104), and serotonin stimulates CRH transcription (105). Jorgensen et al. increased neuronal 5-HT content in the rat by systemic administration of 5-hydroxytryptophan (5-HTP) in combination with the 5-HT reuptake inhibitor fluoxetine. Expression of rCRH mRNA in the paraventricular nucleus (PVN) was increased by 64%; pro-opiomelanocortin (POMC) mRNA in the anterior pituitary lobe was increased by 17% and ACTH secretion was stimulated five-fold (105).

In addition to having a significant affinity for the cortisol receptor, a reciprocal relationship may exist between glucocorticoid and progestin binding sites; therefore, the binding of one class of steroid to its own site results in a decrease in the affinity of the other site for its respective class of steroid (101). Thus, the progesterone receptor complex may indirectly decrease the ability of the cortisol-GR complex to inhibit ACTH secretion.

Progesterone has been shown to be abundantly secreted in response to stress in rodents (106) and there is evidence that stress increases progesterone, at least in the follicular phase of the menstrual cycle of women (107). Salivary concentrations of progesterone were highly correlated with salivary cortisol but the time-course of response to a cold pressor test was different with cortisol reaching high levels at 15 min post stress and progesterone reaching highest levels at 42 min (107). Also, Childs et al. (108) using the Trier Social Stress Test (93), found that women in the follicular phase of the menstrual cycle responded to the stressor with increased concentrations of serum progesterone. At 10 min after completion of the tasks, subjects had an average of 510 pg/ml progesterone while controls had 400 pg/ml (P <0.05). Although not determined, the source of progesterone in this case is most likely the adrenal gland; it is, of course, an intermediate in the biosynthesis of corticosterone and cortisol. This response to low levels of progesterone may also apply to postmenopausal women.

Synthetic progestins may actually promote feedback inhibition of ACTH. Administration of a synthetic progestin (megestrol, 160 mg/day) decreased serum cortisol levels by 76%. This was partly due to a 25% decrease in the CBG (103). The reduction in cortisol would be expected to benefit most breast cancers. Alternatively, administration of the progestin levonorgestrel has been shown to suppress tumor progression in luminal A cell lines via binding to and apparently activating GR but increasing cell proliferation in triple negative breast cancer (TNBC) cell lines (109). Each of the cell lines express the GR gene (110) so direct effect of the GR complex is possible. The mechanism by which the stimulation occurs has not been identified but under some conditions glucocorticoids activate serum and glucocorticoid-induced protein kinase 1 (SGK-1) (111). Under these conditions, glucocorticoids would increase tumor proliferation and suppress the host defense, leading to tumor growth.

These observations require further study to understand the processes observed in this study.

Progesterone is increased in gestation and may be increased with stress. It may act in one of several ways to either antagonize the feedback effect of cortisol on ACTH secretion or to increase ACTH secretion itself through an increase hypothalamic serotonin secretion. Whatever the mechanism, it is the progestin component of hormone postmenopausal therapy that is responsible for the increased risk of breast cancer (112).

### 5.3 Mineralocorticoids

Normally, glucocorticoids induce homodimerization of GRα. However, molecular complexes between the steroid receptor transcription factors may occur resulting in heterodimer formation. The interaction of GR and the mineralocorticoid receptor (MR) is capable of decreasing or otherwise modifying the transcriptional responses to GR as well as its biological roles (113). High expression of cytoplasmic MR has been associated with poor relapse-free survival (RFS) in hormone receptor (HR) positive breast cancers but was associated with better cancer specific survival in TNBC (114). Thus, the presence of elevated MR appears to reverse the positive effect of GR on RFS in HR positive cancers (115) and the negative effect on RFS in TNBC.

Posani et al. (116) have investigated the mechanisms by which MR exerts its effect in TNBC. They chose several cell lines that represent TNBC. Great variability among cases was evident from the difference among four TNBC cancer cell lines. MDA-MB-234 and SUM159 cell lines expressed high amounts of MR on Western blots while MR was barely detectable in MDA-MD-468 and HS578T cell lines. Using those with high MR, addition of either dexamethasone or aldosterone induced formation of heterodimers or other multimers of MR and GR or MR and phospho-Ser145-GR (pGR) according to proximity ligand assays. Alternatively, TGFβ-induced formation of cytoplasmic MR-GR complexes required both p38 MAPK signaling and pSer134-GR. MR antagonists (spironolactone, finerenone) blocked MR-GR and MR-pGR interactions and significantly reduced aldosterone- or TGFβ1-induced migratory and stemness properties in TNBC. In addition, lung metastases were reduced in MR-deficient models following mouse tail-vein injection.

The studies of Posani et al. (116) describe the conditions for activation of complexes of MR and GR but their studies of the effects of the heterodimers are inconsistent with data from patients with TNBC (114). It is evident that interactions of GR with MR, ER, and PR will be needed to understand the magnitude of the effects of the several potential heterodimers on breast cancer.

## 6 GC levels and diurnal variation in relation to breast cancer risk and survival

Researchers have long questioned whether chronic psychological stress with its concomitant elevation of cortisol levels is related to risk of developing breast cancer. Interview-based case-control and prospective cohort studies have produced mixed results. A recent case control study reported a multivariable-adjusted odds ratio of 3.38 (95% CI: 2.56-4.47) for women who reported high versus low stress in the preceding year (117). An earlier meta-analysis of 11 cohort studies judged to have adequate quality concluded that a history of stressful life events was associated with a small increase in breast cancer risk; relative risk = 1.11 (95% CI: 1.03-1. In contrast, two notably large, well-designed cohort studies found no association with breast cancer risk. Butow, et al. (118), in a study omitted from the meta-analysis, followed 2,739 women with family histories of breast cancer for 15 years, repeating assessments every three years, and found no association with risk either for total acute stressful events or chronic stress (118). Schoemaker, et al. (119) studied a cohort of 106,000 women in the U.K., followed over a mean of 6.1 years, and found no consistent associations between risk and the frequency or total number of stressful events experienced in the five years before baseline assessment (119).

The inherent difficulties of measuring stress using self-report without introducing recall, selection, or diagnostic ascertainment biases are obvious. Therefore, biomarker-based studies are thought to provide a more valid approach. Several studies have measured cortisol directly in serum or saliva, or indirectly as a metabolite in blood or urine, to examine associations with breast cancer risk and survival. These studies address both the absolute levels of exposure to endogenous GCs and the patterns of diurnal variation. A metabolomics study among Black women in South Africa of 396 newly diagnosed cases and 1:1 matched controls queried 1,732 serum metabolite features using LC-MS. The three features most strongly associated with breast cancer were cortisol and compounds related to tryptophan metabolism and fatty acid transport (120). After full covariate and false discovery rate adjustment, the odds ratio for cortisol was 1.66 (FDR 95% CI: 1.19-2.31). Prospective cohort studies of the circulating metabolome did not report such an association for metabolites (121, 122):. However, cortisol or its metabolites were not included in the metabolomic investigation. A nested case-control study was established within the European Prospective Investigation into Cancer cohort, which included 1624 first primary incident invasive breast cancer cases (with known estrogen and progesterone receptor and HER2 status) and 1624 matched controls. Metabolites (n = 127, acylcarnitines, amino acids, biogenic amines, glycerophospholipids, hexose, sphingolipids) were measured by mass spectrometry in pre-diagnostic plasma samples and tested for associations with breast cancer incidence using multivariable conditional logistic regression. However, Houghton, et al. conducted a nested case-control study in a cohort with a family history of breast cancer and focused more narrowly on the metabolome covering 36 steroids in urine using GC-MS. They observed the most significant positive risk associations with a group of 14 glucocorticoid metabolites (123). A doubling in these metabolites together had an odds ratio of 2.7 (95% CI: 1.3-5.3). Tumor characteristics were available on 76% of our sample, and 62% of cases were ER+, 49% were PR+, and 46% were HER2+, however given the small overall number of cases, they did not stratify by subtype in analyses.

Wang et al. (124) reported that cortisol levels over a longer time period were associated with survival in non-metastatic breast cancer. Sixty-six postmenopausal patients with estrogen receptor positive cancers were scheduled for postsurgical radiotherapy and adjuvant endocrine treatment. Serum was collected at six different time points - before the start of radiotherapy as baseline, immediately after radiotherapy, and then 3, 6, 12 months, and 7–12 years after radiotherapy. Eight steroid hormone concentrations were measured by LC-MS/MS. Baseline cortisol levels were lower in patients who relapsed than in those who did not (p < 0.05) but during follow-up, cortisol and cortisone concentrations decreased in relapse-free patients, whereas they increased in patients who relapsed. Levels of estrone, estradiol, progesterone, 17α-hydroxyprogesterone, androstenedione, and testosterone did not discriminate between these groups.

Women with advanced breast cancer have been shown to have disrupted circadian rhythms compared to healthy controls based on salivary cortisol (126). Sephton et al. (125), enrolled 104 patients with metastatic breast cancer in a prospective study on the association of diurnal cortisol variation with overall survival (125). Breast cancer subtypes were not recorded in these studies. Salivary cortisol was measured on three consecutive days, at 0800, 1200, 1700, and 2100 hours, and the slope for change in log cortisol concentration was calculated. **Figure 3** shows diurnal cortisol trends from subjects whose cortisol slope was above versus below the median. Although there is an appearance of elevated salivary cortisol in the group with flatter slope, the area under the curve and mean levels were not significantly different between these groups. The important difference is indicated by the much greater variability (approximately 50-fold greater) in the group with flattened profiles than in the group with more normal circadian patterns showing a steady decrease from morning until evening. Subjects without normal diurnal variation had significantly shorter overall survival, and this association remained after adjustment for demographics, treatment received, reported pain, and depression symptoms (Cox proportional hazards, P = 0.004).

**Figure 3:**
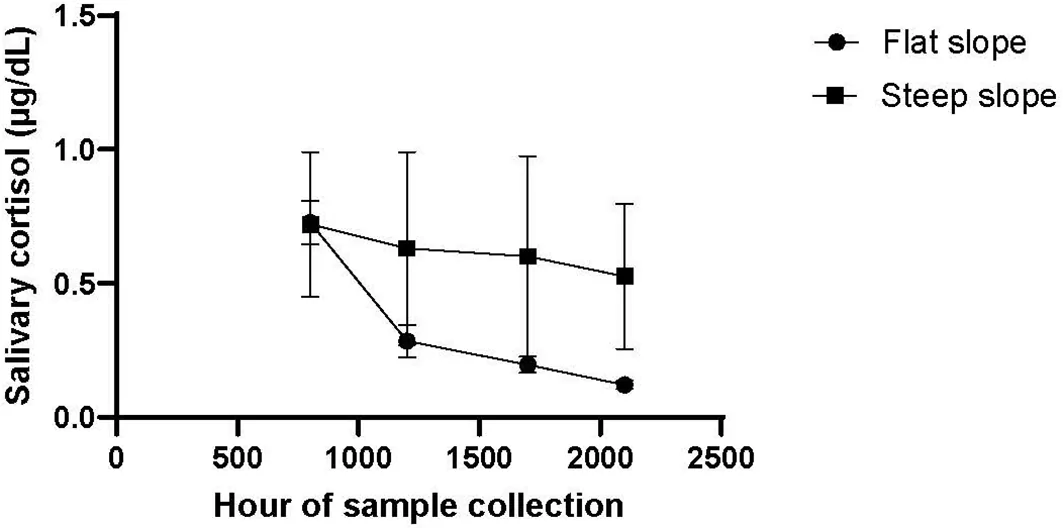
Mean (bars show 95% confidence intervals) of diurnal salivary cortisol levels at four times of day (military time) for two equal groups of patients split at the median diurnal cortisol slope (-0.091 log [µg/dL] per hour). The average value of the diurnal cortisol slope for the flat slope was -0.040 and for the steep slope group it was -1.28 log [µg/dL] per hour. There were 52 patients in each group. The highest value is at 2100 hr. Adapted from Sephton et al., 2000 (125).

In cross-sectional analyses, this study also reported that subjects who lacked a normal diurnal variation had a decreased proportion of circulating NK cells as measured by flow cytometry (P = 0.007), as well as decreased NK cell cytolytic activity, determined by the chromium release method (P <0.05). While these data suggest a possible causal mechanism for the survival association, it is difficult to infer causality due to the study’s observational design and the need for additional validation. Moreover, it remains unclear whether this relationship is causal or instead an epiphenomenon linked to a true causal mechanism.

The association between flattened salivary cortisol patterns and cancer survival was also confirmed in a more recent study of 129 patients with newly diagnosed metastatic renal cell carcinoma who provided five saliva samples per day for three consecutive days had a life expectancy of greater than 4 months (41). Flat cortisol slope remained significantly associated with shorter survival after adjustment for clinical risk categories; hazard ratio = 1.9 (95% CI: 1.3-3.0). Transcriptomic profiling of peripheral blood mononuclear cells suggested that abnormal cortisol regulation might mediate the association observed between depression scores and survival through increased expression of inflammatory signaling in these renal cell carcinoma patients.

Thus, relationships of salivary and serum cortisol to clinical outcome apparently is not limited to metastatic breast cancer.

In sum, information available to date suggests that it is apparent that cancer patients with flattened, highly variable, diurnal cortisol patterns have worse outcomes compared to those in patients with more normal circadian patterns, and that this potentially could be attributed to changes in inflammatory response mechanisms. Furthermore, there is some evidence that salivary and serum cortisol levels are often elevated in the early stages of breast cancer and that relapse after treatment is associated with maintenance of the elevated levels over time.

## 7 Mechanisms by which cortisol modifies cancer risk

### 7.1 Cortisol effects on metabolism

In neoplastically transformed cells, genetic mutations have occurred, resulting in metabolic reprogramming, which enables continuous cell proliferation and survival. Many processes undergo reprogramming. Among them are glucose metabolism, fatty acid metabolism, glutamine metabolism, and pathways that directly affect the tumor microenvironment (127). Cortisol plays an important role in the metabolic changes under conditions in which cortisol secretion is increased. It increases glucose availability through glycogenolysis, gluconeogenesis, lipolysis, decreases in insulin sensitivity, and inhibition of glucose uptake by the skeletal muscle and adipose tissue. The increased availability of glucose provides a substrate for aerobic glycolysis, a process promoted by cancer cells (the Warburg effect). Mechanistically, liganded GR binds to glucocorticoid response elements in the PDK-4 promoter which results in phosphorylation of pyruvate dehydrogenase (PDH), inactivating conversion of pyruvate to acetyl-CoA, forcing pyruvate toward glycolysis and lactate formation.

Elevations in glucocorticoids also may promote elevation in glucosamine, an important source of energy for cancer growth. Glucocorticoids increase blood glucose by stimulating hepatic gluconeogenesis and causing insulin resistance. Increased intracellular glucose availability can increase flux through the hexosamine biosynthetic pathway (HBP), which converts fructose-6-phosphate to glucosamine-6-phosphate. Thus, glucocorticoids may indirectly enhance hexosamine pathway activity via their hyperglycemic activity (128).

Whether an estrogen antagonist will be effective in breast cancer is an open question. A GR antagonist relacorilant has been evaluated in the ROSELLA trial for effectiveness in ovarian cancer. Patients were randomly assigned 1:1 to receive relacorilant (150 mg orally the day before, day of, and day after nab-paclitaxel infusion) plus nab-paclitaxel (80 mg/m2 intravenously on days 1, 8, and 15 of each 28-day cycle) or nab-paclitaxel monotherapy (100 mg/m2 intravenously on the aforementioned schedule). At a median follow-up of 24·8 months (95% CI 23·6–25·7), the addition of relacorilant to nab-paclitaxel resulted in a statistically and clinically significant improvement in overall survival compared with nab-paclitaxel monotherapy (hazard ratio for death 0·65 [95% CI 0·51–0·83]; p=0·0004); 18-month overall survival was 46% and 27%, respectively (129).

Ovarian cancer is not uniformly estrogen-dependent, but estrogen signaling contributes to the development and progression of a majority of ovarian cancers (130). The ovarian cancer subtypes that reliably show estrogen-driven proliferation are endometrioid ovarian carcinoma and high-grade serous carcinoma (HGSC). However, antiestrogen treatment alone has been shown to be largely ineffective (131). Evidently, ER+ ovarian cancer differs from ER+ breast cancer in that it is less dependent on estrogen. Treatment with the GR antagonist does not promote progression of ovarian cancer as would be expected for ER+ breast cancer.

In breast cancer antagonism of GR should reduce the suppression of the immune responses to cancer and should reduce the glycolytic effects that promote tumor growth. For TNBC this would promote a positive outcome. However, GR expression in ER-positive breast cancer is associated with a better prognosis as a result of ER-GR crosstalk (115). While a GR antagonist may alleviate the immunosuppression and increased glycolytic availability in ER+ breast cancer, a GR antagonist would reduce the suppression of estrogenic activity associated with GR. The mechanisms by which cortisol modifies cancer risk are summarized in **Table 1**.

**Table 1:** Comparison of glucocorticoid functions in normal and breast cancer cells.

Comparison	Normal	Breast cancer
Primary function	Antiinflammatory Immunosuppressive hyperglycemic	Survival Metabolic colonization Metastasis
Cell cycle	Growth suppression	ER+ cells: growth suppression TNBC: survival, chemoresistance
Metabolic effects	Homeostatic	Hypoglycemia PDK-4 driven glycolytic shift Glucosamine elevation
Stress Integration	Restores homeostasis	Amplifies metabolic programs Activation of SGK-1
Immune effects	Immunosuppression	Immune evasion Inhibition of lymphocytes into TME Promotion of PD-1

### 7.2 Interaction of cortisol with IGF-I

Cortisol inhibits growth hormone-promoted transcription of IGF-I, and immunoreactive IGF-I levels in fibroblast cultures decrease in response to glucocorticoids (132). The converse is also true. Early work demonstrated the role of growth hormone (GH) in opposing the effect of cortisol on leukocytopoiesis. Homogenates of the anterior pituitary, but not of the posterior pituitary or skeletal muscle, prevented cortisol-induced leukopenia in hypophysectomized, adrenalectomized rats. Growth hormone of rat origin (NIH**-** GH-S9) also opposed cortisol-induced leukopenia in hypophysectomized rats (133). Other studies have shown stimulation of DNA synthesis in human T-lymphocytes by growth hormones (134) and enhancement of cytotoxic activity of human natural killer (NK) cells (135). This action is mediated by the local production of IGF-I. Growth stimulation is completely inhibited by preincubation with either an antibody to the IGF-I receptor or IGF-I itself (136). The relationship is complex because growth hormones stimulate the formation of IGF-I. Administration of GH may result in GH-receptor activation or secondarily may increase IGF-I transcription; both may stimulate kinase pathways, e.g., phosphoinositide 3-kinase (PI3K) and MAPK. While IGF-I acts to counteract the lymphocytolytic action of cortisol, cortisol has been shown to be necessary for the ontogeny of pituitary somatotrophs (137). According to Basu et al. (138) information is emerging in several organ systems that support the role of GH-regulated inflammation in cancer. Overall, it is evident that autocrine- or paracrine-derived GH can potentially exacerbate local inflammation in the tumor microenvironment.

### 7.3 Stimulation of catecholamine synthesis and activity by adrenal steroids

Stimulation of the adrenal gland by ACTH results in increased concentrations of cortisol, DHEA, and DHEA sulfate within the adrenal cortex and medulla. Increasing concentrations of DHEA sulfate stimulate norepinephrine biosynthesis in the adrenal medulla by increasing both mRNA and protein levels of tyrosine hydroxylase (46). It also promotes trafficking of catecholamine vesicles (46). In addition, cortisol, which is present in high concentrations in blood traversing the medulla, increases the expression of phenylethanolamine N-methyltransferase, which is essential for the conversion of norepinephrine to epinephrine, the main catecholamine released from the adrenal medulla in response to stress (139).

In addition, glucocorticoids increase plasma levels of catecholamines by inhibiting their extra-neuronal uptake and induce super-sensitivity to catecholamines in the heart and other organs by upregulating components of the beta-adrenoceptor signal transduction system (140, 141). Thus, stressors that activate the HPA axis also increase catecholamine secretion, increase blood levels of catecholamines by inhibiting uptake, and increase the response to catecholamines in some tissues.

### 7.4 Effects of cortisol on the immune system

The glucocorticoid receptor is ubiquitously expressed in human tissues and organs, including neural stem cells and lymphoblasts. GR functions as a hormone-dependent transcription factor that regulates the expression of glucocorticoid-responsive genes, which represent 3-10% of the human genome. Glucocorticoids can cause neutrophilic leukocytosis, eosinopenia, monocytopenia, and lymphocytopenia. Cortisol brings about neutrophilic leukocytosis by inhibiting the function of neutrophil adhesion molecules (e.g., L-selectin, integrins). Blocking the adhesion molecules prevents trans-endothelial migration, keeping neutrophils in circulation and away from inflamed tissues. Although neutrophils are major sources of pro-inflammatory cytokines, reactive oxygen species, and proteases, without tissue infiltration they are unable to amplify local inflammation or contribute to tissue or tumor damage. Granulocytes are minimally affected but monocyte-macrophage function is sensitive to glucocorticoids. GC administration causes transient lymphocytopenia in all detectable lymphocyte subpopulations, particularly recirculating thymus-derived lymphocytes. The mechanism of lymphocytopenia is probably the redistribution of circulating cells to other body compartments (142).

.Ligand-bound GRs act as potent immunosuppressive agents that interfere with binding of AP-1 and Nf-κB to their DNA binding sites, resulting in decreased transcription of inflammatory cytokines such as the interleukins IL-1, IL-6, IL-8, and TNFα. And they promote an anti-inflammatory Th2 reaction by inhibition of IFN-γ and stimulation of IL-4, IL-10 and TGF-β secretion (122). Thus, cortisol elevation acts to suppress availability of neutrophils to the antigen-expressing cells, suppresses the circulation of Th1 cells, inhibits Th1 cytokine secretions, and increases the transcription of anti-inflammatory cytokines (**Table 2**).

**Table 2:** Effect of cortisol on cellular immunity.

Component	Mechanism	Effect
Neutrophils (+)	Inhibition of adhesion molecules	Limits tissue access
Lymphocytes (-)	Redistribution to other organs	Decreased circulatory abundance
Monocytes (-)	GRE binding to AP-1 and NF-κB (-)	Decreased inflammatory cytokines
Eosinophils (-)	GRE binding to AP-1 and NF-κB (-)	Less inflammation, increased IL-4

The symbol (-) indicates decrease, (+) indicates positive effect.

#### 7.4.1 Effect of GC on lymphocyte activation

Glucocorticoids enhance cytokine production of regulatory T cells (Tregs) by upregulation of FOXP3 expression (143). Tregs are a specialized subpopulation of T cells that play a key role in maintaining tolerance to self-antigens and in suppression of excessive immune responses after antigenic stimulation (144). In addition, glucocorticoids inhibit early steps of lymphocyte activation by stimulation of cAMP (145). Indeed, treatment of resting human lymphocytes with dexamethasone increases prostaglandin E2-stimulation of cyclic AMP accumulation in a time- and concentration-dependent manner.

Treatment with the glucocorticoid agonist dexamethasone has no significant effect on adrenoceptor density, or immunodetectable G-proteins, and pertussis toxin substrates are not affected (145, 146). It was concluded in these studies that dexamethasone treatment promotes cAMP formation in resting human lymphocytes by altering the adenylyl cyclase enzyme activity rather than G-proteins or hormone receptors. Thus, glucocorticoid exposure results in enhanced capability of cyclic AMP-generating agonists to inhibit the early steps of lymphocyte activation and lymphocyte proliferation.

#### 7.4.2 Effect of cortisol on tumor-infiltrating lymphocytes

Cortisol may have a significant effect on the transfer of tumor-associated lymphocytes (TALs) or tumor-infiltrating lymphocytes (TILs) to antigen-bearing tumor cells. The activation of the intercellular adhesion molecule-1 (ICAM-1) gene by TNFα from macrophages is an essential step in early inflammatory events, leading to the recruitment of leukocytes to the site of inflammation. TNFα activates transcription of the ICAM-1 gene via the NF-κB signaling pathway in the human ICAM-1 gene promoter region (147). Glucocorticoids suppress ICAM-1 transcription by the ability of ligand-bound GR to interfere with NF-κB signaling at the NF-κB binding site without increasing IkBα expression. According to Liden et al. (148) the conformational change observed at the NF-kB binding site of GR may be controlled either by direct binding of the GR to the NF-κB complex or by GR-induced recruitment or displacement of other proteins.

#### 7.4.3 The tumor microenvironment

The microenvironmental stroma contributes to host defense. In a dermatological study by Boxman et al. (149), fibroblasts exposed to conditioned media derived from cultures of normal human keratinocytes or squamous carcinoma cells (SCC-4) produced elevated levels of both IL-8 and IL-6 mRNAs, as well as their respective proteins. Formation of these inflammatory cytokines are inhibited by cortisol. Additional studies have shown that IL-1α derived from keratinocytes induced the proliferation of fibroblasts and the secretion of IL-6 and IL-8 in a concentration-dependent manner (149–151). These pro-inflammatory interleukins promote cancer progression. IL-6 modulates immune cells, stromal cells and endothelial cells to support tumor growth (152). IL-8 recruits neutrophils and myeloid-derived suppressor cells (MDSCs) and enhances epithelial to mesenchymal transition (EMT) (153). Stromal cells add to the protumor environment by their ability to express the GR. These include: fibroblasts, macrophages, adipocytes, and immune cells. Gene expression profiling studies have compared tumor-associated stroma to normal stroma and have shown that cancerous cells can dramatically increase stromal GR mRNA expression (154, 155).

GR expression is increased significantly with grade in stroma isolated from paraffin-embedded tissues (**Figure 4**), whereas progesterone receptor (PR) expression is significantly decreased compared to that in the control stroma (155) (not shown). A combination of increased GR expression and elevated concentrations of circulating cortisol act to suppress the local immune response and promote tumor cell proliferation.

**Figure 4:**
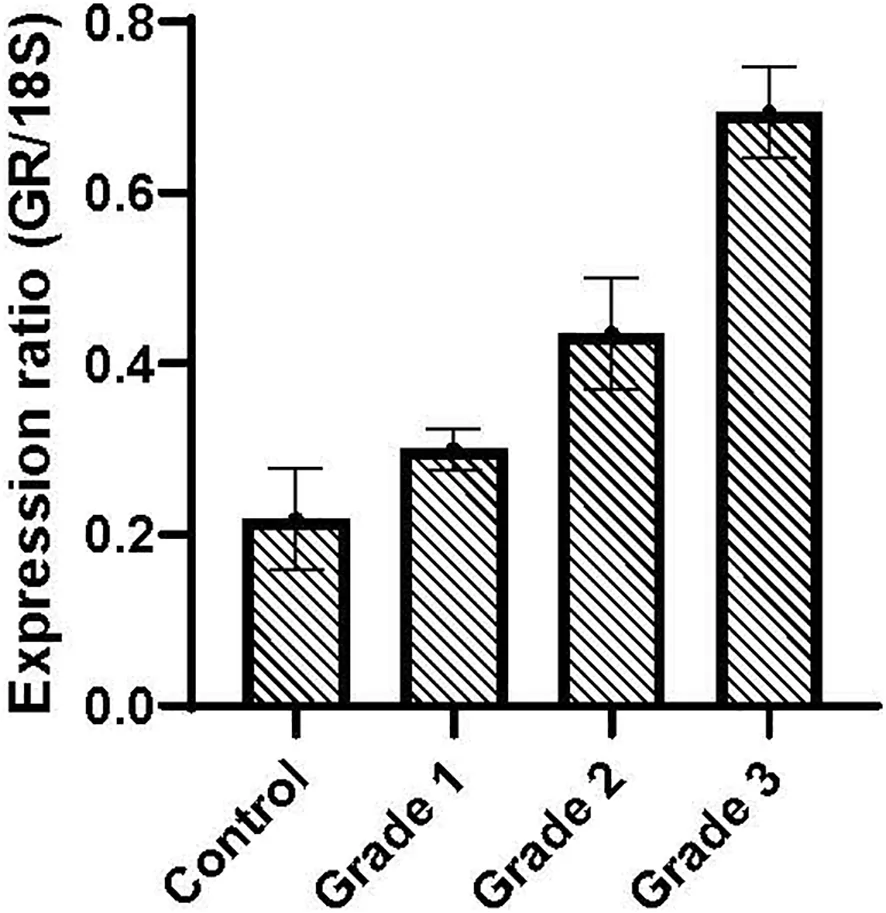
Expression of GR in stroma derived from different tumor grades. Grade 2 was significantly different from control (P = 0.05) and Grade 3 was significant from control (P = 0.001). Adapted from Smith et al., 2007 (155).

To oppose the production of inflammatory cytokines in the stroma, liganded GR directly interacts with NF-κB, preventing it from binding to DNA, reducing the transcription of pro-inflammatory genes (156). GR activation also leads to the expression of IκB, an inhibitor that prevents NF-κB from translocating to the nucleus (157). As a result, in the tumor microenvironment, glucocorticoids suppress the production of the pro-inflammatory cytokines IL-2, TNFα, and IFNγ, and induce the secretion of IL-10.

In addition, elevation of GC dramatically induces immune checkpoint receptors, including PD-1, Tim3, and Lag3, thereby protecting the tumor from TILs. On the other hand, loss of GR potentiates the response to immune checkpoint expression (54). Analysis of RNA profiles showed that NR3C1, the gene encoding the glucocorticoid receptor, was most highly expressed in the Tim-3-PD-1+ CD8+ and Tim-3+PD-1+ CD8+TIL subsets, which contain effector and terminally dysfunctional CD8+ TILs, respectively (158). Thus, cortisol may protect tumor cells from TIL, allowing the tumor to progress.

The high levels of GR observed in breast cancer (155) relates to the findings reported by Matossian et al. (159) and the observation that high GR expression is associated with poor prognosis in women with ER negative breast cancer. In a recent study patients with untreated primary triple negative breast cancer (TNBC) a significantly higher percentages of immunosuppressive tumor-infiltrating FOXP3+ and BATF3+ cells were found in tumors with high levels of GR compared to those with low levels of GR suggesting that GR expression is associated with an immunosuppressed microenvironment (34). Thus, locally produced GR in the microenvironment along with endogenous cortisol results in an environment in which the host defense is compromised.

On the other hand, reducing local steroidogenesis appears to reduce TNBC progression via suppressing GC signaling (160) suggesting that GCs promote TNBC proliferation. On the other hand, in women with ER+ tumors interaction of liganded GR with the ERE correlates with improved relapse-free survival in patients (161, 162). This is consistent with the studies described earlier in this review in which TNBC cells cultured *in vitro* were stimulated by levonorgestrel a synthetic steroid which binds to and activates the GR (109).

Currently, glucocorticoids are commonly used in conjunction with immune checkpoint inhibitors, but the pros and cons of glucocorticoids as immunomodulators in cancer immunotherapy remain to be determined (163, 164).

#### 7.4.4 Effects of cortisol acting through macrophages

Macrophages are essential components of both innate and adaptive immunity and play a central role in host defense and inflammation. Activated macrophages are divided into two subsets, (M1) and (M2). The M1 phenotype is characterized by the expression of high levels of pro-inflammatory cytokines, including IL-12, IL-23, and tumor necrosis factor-α (TNFα), as well as high nitric oxide and reactive oxygen intermediate production. In contrast, cells of the M2 phenotype typically produce IL-10 and the IL-1 receptor antagonist (IL-1Ra), and have high levels of scavenger, mannose, and galactose receptors.

The phenotypes of polarized M1 and M2 macrophages can be reversed *in vitro* and *in vivo* by cytokines and growth factors. Interferon-c (IFN-c) induces M1 macrophages, whereas stimulation of macrophages with IL-4 or IL-13 induces M2 macrophages (165).

Cortisol promotes polarization of macrophages toward the M2 phenotype, which is involved in the anti-inflammatory response. Glucocorticoids also have profound effects on the antigen presentation to T cells by macrophages. Corticosteroid drugs inhibit the expression of surface I-region-associated (Ia) antigens by peritoneal macrophages both *in vitro* and *in vivo*, reduce the production of IL-1, and inhibit antigen presentation for T cell proliferation by macrophages. In one study the doses of cortisol or prednisolone, which inhibit 50% Ia expression in cultured macrophages, ranged from 2 to 5 x 10–^8^ M and were well within the range of endogenous hormones (166).

In support of the immunosuppressive activity cortisol on macrophages, cortisol upregulates HSD11β type 1 in macrophages (58, 59), T- and B-cells, and dendritic cells (60, 61), thus maintaining the active form of cortisol for its role in promoting the M2 macrophage phenotype.

### 7.5 Cortisol acting through corticosteroid binding globulin

Binding of cortisol by CBG plays an important part in limiting availability of cortisol to extravascular compartments. It also acts when bound to plasma membranes as a prohormone which is activated by the binding of cortisol. As such the cortisol-CBG complex activates adenylyl cyclase in liver and cancer epithelial cells resulting in accumulation of cAMP within these cells.

Binding of cortisol to CBG is reduced reversibly by pyrexia and acidemia, and reduced irreversibly by neutrophil elastase (NE) cleavage, converting high cortisol-binding affinity CBG to a low affinity form. These characteristics allow for the targeted delivery of cortisol to tissues at the time and body site where cortisol is required such as in sepsis and septic shock (1) and in metabolic changes associated with anorexia nervosa (167). In addition, high titer inflammatory cytokines suppress CBG hepatic synthesis, increasing the serum free cortisol fraction.

CBG is a 50–60 kDa glycoprotein that binds cortisol at a 1:1 molar ratio with a Ka of 76 x 10^6^ L/mol; it also binds progesterone with a Ka of 24 x 10^6^ L/mol (1). About 80 percent of cortisol in the circulation is bound to CBG and 15% to albumin. The remainder is free and capable of diffusing across plasma membranes. The plasma concentration of CBG the liver is increased by estrogen in oral contraceptives or by Premarin or diethylstilbestrol by as much as 2 to 3-fold (168) and is suppressed by glucocorticoids (169). However, at a cellular level neither estradiol, testosterone, nor dexamethasone alters the production of CBG by HepG2 cells (170, 171) indicating an indirect process. CBG is produced by the kidney and other organs to some extent but this is unlikely to be the source of the increase due to estrogen administration. An effect on CBG biosynthesis by IL-6 has been shown in HepG2 cells (172). When Hep G2 cells were exposed to different concentrations of IL-6 for variable time intervals, IL-6 caused a dose- and time-dependent decrease in the amount of [^35^S]methionine-labeled CBG immuno-precipitated from the culture medium. This effect could be greatly reduced by preincubation of IL-6 with its neutralizing antibody and reversed by removing the cytokine from the culture medium. This potentially will decrease the total binding capacity of CBG in plasma and increase the availability of free cortisol in situations of prolonged stress.

Another way that CBG interacts with cortisol is in binding to hepatic plasma membranes. Scatchard analysis of the soluble receptor at 37 C showed a single set of high affinity binding sites, with a Kd of 44 nM and a binding capacity of 7.3 pmol/mg protein. The association rate constant (k1) was 0.92 x 10^5^ M-1 min-1 at 37 C, and the dissociation rate constant (k2) was 1.0 x 10–^3^ min-1. Only unliganded CBG was able to bind to the plasma membrane receptor. Steroids that bind to CBG, e.g. corticosterone, cortisol, and progesterone noncompetitively inhibit binding of CBG to its receptor (173). Under normal conditions CBG would not traverse the endothelium. However, binding of cortisol to the previously liganded CBG on the breast cancer cell line MCF-7 results in the induction of adenylate cyclase activity and the accumulation of cAMP cells (174), **Figure 5**.

**Figure 5:**
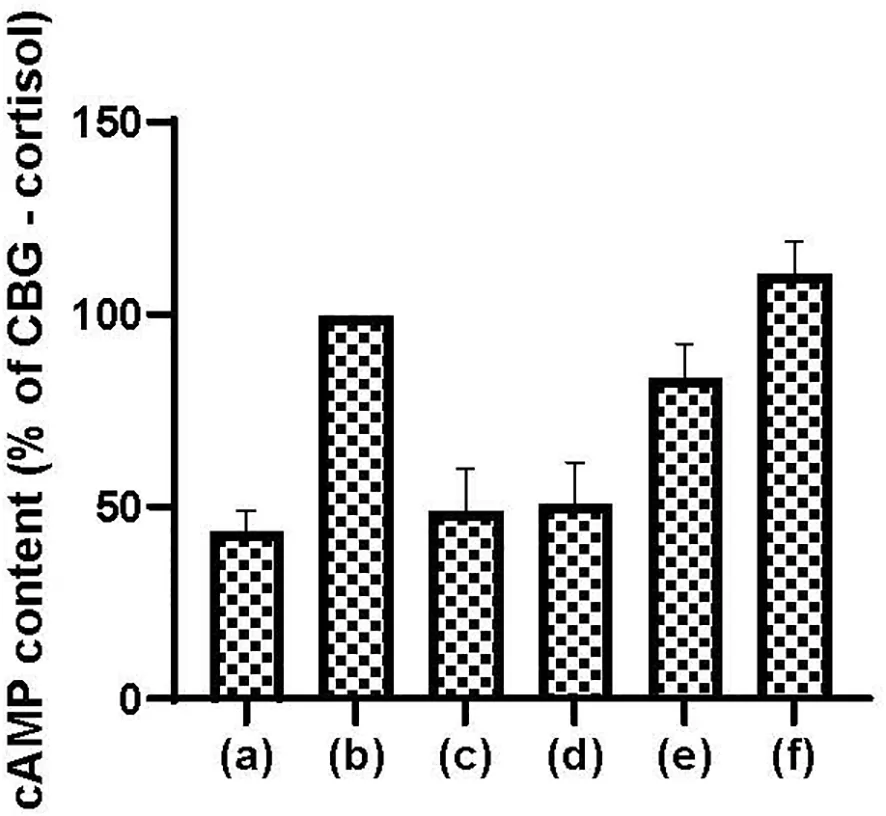
Effect of different molecular forms of CBG and of cortisol on cAMP accumulation in MCF-7 cells. a, control; b, CBG-bound cortisol (1 µM); c, stripped CBG (1 µM); d, cortisol (1 µM); e, regenerated CBG-bound cortisol (1 µM); f, CBG-bound progesterone (1 µM). Adapted from Nakhla et al., 1988 (174).

The data are consistent with the hypothesis that CBG is a prohormone which is activated when cortisol becomes bound to it. In hepatocytes activation of adenylyl cyclase produces cAMP which results in glycogenolysis and inhibition of glycogen biosynthesis, a process that may provide initial glucose substrate for cancer growth.

In the breast cancer epithelium a function that may be relevant is an effect on phosphorylation of junctional proteins (e.g., claudins, occludins), reducing epithelial barrier permeability (175). How cortisol-induced expression of TJ proteins is involved in cell proliferation, transformation, and metastasis is not well understood (66). Nevertheless, experimental evidence suggests that TJ proteins are important players in the initiation and progression of cancer.

In lymphocytes the process is quite different. Maintenance of the CBG-cortisol complex is dependent on a correct cellular organization of the tissue, which is largely dependent on stromal-derived chemokines (176). In lymphocytes membrane-bound CBG inhibits cAMP-dependent protein kinase A (PKA) (146) by blocking Ras activation (177), and by increasing the production of anti-inflammatory IL-10 (178). Thus, cortisol may act through CBG binding to inhibit host defense by suppressing lymphocyte proliferation and increasing anti-proliferative cytokines, thus protecting the tumor from the immune system.

## 8 Effects of glucocorticoids on cancer therapy

An interesting question is how glucocorticoids interact with or modify the effects of breast cancer prevention and cancer treatment regimens. Liang et al. (179) in a recent review discuss how dexamethasone may delay tumor progression by suppressing pro-inflammatory cytokine release, modulating T-cell function, and inhibiting angiogenesis. They also describe how dexamethasone can promote the formation of an immunosuppressive tumor microenvironment by activating the glucocorticoid receptor signaling pathways, thereby accelerating breast cancer metastasis. The role of glucocorticoids in upregulation of ROR-1 kinase and multiple members of the Wnt and Hippo pathways in relation to metastasis have been described in breast cancer cell lines (180).

A primary source of factors affecting growth and differentiation of TNBC cells is the TME. The TME has distinct features compared to the environment of non-cancerous cells. The distinguishing features include low pH, excessive free radicals, and fluctuations in oxygen and nutrient availability. This environment and associated TME factors are known to be potent promoters of cell survival, proliferation, and transformation of cells into a malignant phenotype. The stromal cells of TME exhibit both tumorigenic and anti-cancerous properties. For example, immune cells such as CD8+ T cells and NK cells possess tumor-suppressing activities, while cancer-associated fibroblasts (CAFs) have been implicated in promoting cancer progression and metastasis (181).

TGF-β is a cytokine secreted by fibroblasts, macrophages, and epithelial cells and functions in a tissue-specific and context-dependent fashion within the TME of TNBC. TGF-β1 is specifically involved in immune suppression, angiogenesis, apoptosis, cell growth, and epithelial to mesenchymal transitions (EMT) during development and metastatic cancer progression (162, 182). TGF β1-mediates activation of p38 MAPKs and subsequent post-translational modification of unliganded GR, specifically by phosphorylation of GR Ser134 (183).

Several genes have been identified as crucial factors for GR-driven actions in TNBC, and many of these genes are important for metastatic cancer processes including chemotherapeutic resistance. SGK-1 and MKP-1 are among such GR activated genes whose expression promotes resistance to taxane-based chemotherapy such as paclitaxel or doxorubicin in breast cancer models (184). Physiologic cell stress such as hypoxia promotes p38 MAPK activation and phosphorylation of GR at serine 134 which results in sustained protein tyrosine kinase-6 (PTK6) induction via a feed-forward signaling mechanism.

Among GR-target genes, PTK6 has been significantly implicated as an effector of GR signaling in TNBC models. PTK6 is an intracellular soluble tyrosine kinase whose structure is closely related to the c-Src kinase. Primary TNBC tumor explants and tumor cell lines show a HIF1/2-dependent robust induction of PTK6 mRNA and protein upon treatment with dexamethasone. PTK6 enhances signaling downstream of EGFR and HER2. It activates STAT3 and STAT5B transcriptional programs and stimulates cell cycle progression via inactivation via phosphorylation of p27/CDKN18 (181).

### 8.1 Effect of glucocorticoids on DNA damage response therapy

Sriramulu et al. (185) describe clinical trials that focus on DNA repair mechanisms after radiation exposure. It is expected that target-based radio-sensitization approaches would increase the effectiveness of radiotherapy by selectively sensitizing tumor tissue to ionizing radiation (186). Recently, a variety of approaches have been used to develop radiosensitizers that are highly effective and have low toxicity (187). A number of molecular targets that are sensitive to radiation killing are being studied. These include ATM, TR, CDK1, CDK4/6, CHK1, PARP1, WEE1, and MPS1/TTK. The drugs that are being studied include inhibitors of PARP1 (Olaprib and Niraparib) and inhibitors of HER2, EGFR, VEGF, and mTOR. The effect of glucocorticoids on these systems has not been studied directly. However, the evidence is that elevations of stress hormones will facilitate the process of DNA damage in cells exposed to radiation. Flint et al. (188) have shown that dexamethasone or epinephrine increase the rate of damage in 3T3 cells manyfold. They further showed that anti-progestin RU486 is able to reverse the effects of the glucocorticoid, and that propranolol will reverse the effects of epinephrine. The mechanism may involve an effect of stress hormones on reactive oxygen species (ROS) and reactive nitrogen species (RNS). In experiments of Flaherty et al. (189) acute exposure to cortisol and NE significantly increased levels of ROS/RNS and DNA damage and this effect was diminished in the presence of receptor antagonists. Cortisol induced DNA damage and the production of ROS was further attenuated in the presence of an iNOS inhibitor. An increase in the expression of iNOS in response to psychological stress was observed *in vivo* and in cortisol-treated cells. Thus, elevations in glucocorticoids will likely make the inhibitors more effective in cell killing. This is counter to the general effect of glucocorticoids on promotion of tumor proliferation. MCF-7 and MDA-MB-231 cell lines responded to cortisol with different time courses with the TNBC line responding more rapidly. The overall effect was not different for most ROS and RNS-inducing agents.

Hormone receptor (HR) positive breast cancers respond differently to GR (161) than HR negative cancers (190). In early stage HR-negative breast cancer high glucocorticoid receptor (GR) expression is associated with increased cancer cell proliferation and with shortened relapse-free survival (RFS) (190). It was observed that cells derived from an adjuvant chemotherapy-treated Discovery cohort with a GR signature that contained GR activation-associated genes was found to predict the probability of relapse. A separate validation study confirmed this observation in ER-negative cancer (Hazard ratio = 1.9; P = 0.012) (190). Although many of the factors by which the liganded GR stimulates cancer progression are not known, the immunosuppressive activity of GCs and the altered metabolism resulting in increased availability of glucose and glucosamine among other factors probably contribute to tumor growth in HR-negative tumor cells as described earlier in this review.

On the other hand, GCs have the opposite effect on cancer cell proliferation in HR+ breast cancer. Glucocorticoids are associated with slowed ER-mediated proliferation in ER+ breast cancer cell lines and longer RFS in breast cancer patients (161). It is not immediately clear why the presence of estrogen dependency should reverse the expected effects of glucocorticoids. However, GR liganding with either a pure agonist or a selective GR modulator (SGRM) slowed estradiol (E2)-mediated proliferation in ER+ breast cancer models. SGRMs that antagonized transcription of GR-unique genes both promoted GR chromatin association and inhibited ER chromatin localization at common DNA enhancer sites. Further, Porter et al. (191) found that SGRMs that antagonized transcription of GR-unique genes both promoted GR chromatin association and inhibited ER chromatin localization at common DNA enhancer sites. Gene expression analysis revealed that ER and GR co-activation decreased proliferative gene activation (compared to ER activation alone), specifically reducing CCND1, CDK2, and CDK6 gene expression. Thus, the interaction of liganded GR with ER results in inhibition of estrogen-promoted cell proliferation in estrogen-dependent breast cancer cells. This is the inverse of the interaction of GR with the androgen receptor (AR). These transcription factors recognize similar DNA motifs and GR can substitute for AR, increasing the potential for prostate cancer proliferation (192).

### 8.2 Effect of glucocorticoids on immune checkpoint inhibitor therapy

According to Acharya et al. (54) active glucocorticoid signaling is associated with failure to respond to immune checkpoint blockade in both pre-clinical tumor models and melanoma patients. Although the immune system has the capacity to fight cancer, signals present within the tumor microenvironment (TME) actively suppress anti-tumor immune responses. In particular, CD8+ T cells, key mediators of anti-tumor immunity, undergo altered effector differentiation that culminates in the development of a dysfunctional or “exhausted” state (193, 194). Dysfunctional CD8+ T cells exhibit defective cytotoxicity, pro-inflammatory cytokine production, and induction of the immunosuppressive cytokine interleukin (IL)-10 (195). Among potentially responsible factors, NR3C1, the gene encoding the glucocorticoid receptor (GR), was found to be the most highly expressed in terminally dysfunctional Tim-3+PD-1+ CD8+ TILs. A gradient of increasing GC signaling from naive to dysfunctional CD8+ TILs was identified (54). They found that repeated activation in the presence of GC profoundly suppressed the production of the pro-inflammatory cytokines IL-2, TNF-α, and IFN-γ, and induced the immune suppressive cytokine IL-10, a phenotype consistent with dampened effector function. Additionally, they found that GC treatment dramatically induced immune checkpoint receptors, including PD-1, Tim-3, and Lag-3, but not Tigit in both murine and human CD8+ T cells. The observed effects of GC were not due to reduced T cell survival or altered proliferation. Thus, glucocorticoids decrease functionality of TILs and interfere with the effectiveness of immune checkpoint blockade.

### 8.3 Effect of glucocorticoids on retinoid drug therapy

Brown et al. (196) reviewed targeted therapy for breast cancer prevention. Among compounds being evaluated were inhibitors of vitamin D and retinoid receptors. For example, Hidalgo et al. (197) found that dexamethasone increased vitamin D receptor (VDR) mediated transcription in the human CYP24A1 promoter. Both VDR protein levels and ligand binding were increased in squamous cell carcinoma in a time-dependent manner. The effect was steroid specific and not produced by dihydrotestosterone.

Bagnoud et al. (198) studied the relationship between GR and VDR signaling. They found that vitamin D (VitD) reduces experimental autoimmune encephalomyelitis disease severity in wild-type (WT) but not in T cell-specific glucocorticoid GR-deficient mice (GR(lck). Mice lacking the GR have diminished VitD-enhanced T cell apoptosis and T regulatory cell differentiation *in vitro* in CD3+ T cells. Mechanistically, VitD does not appear to signal directly via the GR, as it does not bind to the GR, does not induce its nuclear translocation, and does not modulate the expression of two GR-induced genes. However, they observed that VitD enhances VDR protein expression in CD3+ T cells from WT but not GR(lck) mice *in vitro*, that the GR and the VDR spatially co-localize after VitD treatment, and that VitD does not modulate the expression of two VDR-induced genes in the absence of the GR. The data suggest that a functional GR, specifically in T cells, is required for the VDR to signal appropriately to mediate the therapeutic effects of Vit D.

These data indicate that the GR-GC complex is essential for VDR signaling. An increase in GR-GC may assure the activity of VDR. Because RXR requires VDR as an obligate partner to bind DNA for regulation of transcription, increases in VDR may enhance the ability of drugs like IRX4204 and LG100268 to bring about programmed cell death of cancer cells. Thus, in the pharmacologic setting, glucocorticoids act to facilitate the treatment effect. However, the negative effects of GCs on the immune system may obviate the benefit.

### 8.4 Effect of glucocorticoids in PARP1-related drug therapy

Promising agents are currently being tested for prevention of triple negative breast cancer. Among them are poly(ADP-ribose) polymerase inhibitors (196). PARP1 has a role in breast cancer, mediated by its interaction with the progesterone receptor (PR) (15). Initially PARP1 was discovered as part of a protein complex that could interact *in vitro* with ligand-activated PR and assist in DNA binding (199). PARP1 activation also leads to a global increase in poly(ADP-ribose (PAR) levels, modifying histones, transcription factors (TF), nuclear enzymes and nuclear structural proteins. Parylation of histones regulates chromatin structure (200) and recruits DNA repair proteins such as p53, and has also been reported to parylate and modify the function of numerous transcription factors, including AP1, NF-κB, CTCF, and YY1 (201).

At least six new PARP1 inhibitors have been evaluated including fluzoparib and pamiparib (202). But because of resistance to PARP1 inhibitors, dual-target inhibitors that greatly improve the antitumor effect of PARP1 inhibitors, reduce toxicity, and evade resistance by synergistically targeting multiple targets have been developed. Combinations of PARP1 inhibitors and BRD4, PI3K, HDAC, PD-1/PD-L1 inhibitors among others have been evaluated.

There is much evidence that GCs consistently counteract the effect of cytotoxic agents, reducing cell death (203). The mechanism by which GCs confer protection has not been fully elucidated. However, PARP1 and GR act in opposition to each other regulating transcription factor activity such as NF-κB and AP1. While PARP1 promotes NF-κB and AP1 (201), GC treatment induces the downregulation of the survival factor NF-κB and increases FAS and FASL leading to caspase 8 activation and apoptosis.

Inhibitors of PARP should oppose recruitment of enzymes of DNA damage repair and oppose activation of PR. GCs should facilitate the effect of PARP inhibitors by increasing the rate of DNA damage and suppressing transcription factors activation (4). Inhibitors of PARP1 therefore will be promoted by the effect of glucocorticoids. Both will inhibit NF-κB and AP1 and both will oppose DNA repair. Nevertheless, the dose and duration of GCs probably should be limited because of their effects on the immune and other organ systems.

## 9 Summary and conclusions

1. Glucocorticoids are regulated by input form the nervous system, and negative feedback on ACTH secretion is suppressed by feedback from several sources including DHEA and β-arrestin, and progesterone may stimulate ACTH secretion via stimulation of hypothalamic serotonin.
2. Glucocorticoids are produced in several organ sites in addition to the adrenal cortex including the immune system, and peripheral activity is further regulated by 11β-oxidoreductases.
3. Glucocorticoids are often dysregulated in metastatic breast cancer and other chronic disease states with increased variability and increased peripheral concentrations.
4. Ligand-bound glucocorticoid receptors modify metabolism in a manner that supports cancer by increasing glucose and glutamine availability, and under some conditions may directly stimulate cancer cell proliferation.
5. Glucocorticoid bound to its intracellular receptor blocks transcription of Nf-κB and AP-1, reducing inflammatory cytokines and the antitumor immune activity. They also bind to membrane-bound CBG, activating adenylyl cyclase in T cells, suppressing proliferation.
6. Glucocorticoids also modify the availability of immune cells to the tumor in part by suppressing ICAM-1 and by increasing the expression of immune checkpoint receptors such as PD-1 and Tim3, promoting Treg cell proliferation.
7. Glucocorticoids are opposed by effects of DHEA and DHEA sulfate on metabolism and on the immune system.
8. Glucocorticoid agonists may provide benefit in retinoid therapy, PARP1 therapy, and therapy in ER+ cancers but oppose therapy involving DNA damage and immune checkpoint blockade.

## Glossary

HSD11β: 11β-hydroxysteroid dehydrogenase
5HT: 5-hydroxytryptophan
ACTH: adrenocorticotropic hormone
AR: androgen receptor
AVP: arginine vasopressin
BMI: body mass index
PKA: cAMP-dependent protein kinase A
CAFs: cancer associated fibroblasts
CBG: corticosteroid binding globulin
CRH: corticotropic hormone
CRH-R: corticotropic hormone receptor
cAMP: cyclic adenosine monophosphate
DHEA: dehydroepiandrosterone
DST: dexamethasone suppression test
DHEAS: DHEA sulfate
EMT: epithelial to mesenchymal transition
E2: estradiol
FDR: false discovery rate
CLS: crown-like structures
GR: glucocorticoid receptors
NR3C1: glucocorticoid superfamily
SGK-1: glucocorticoid-induced protein kinase 1
GCs: glucocorticoids
GPCRs: G-protein coupled receptors
GR(lck: GR-deficient mice
GH: growth hormone
GREs: GR-responsive elements
HR: hormone receptor
HPA: hypothalamic-pituitary-adrenal axis
IEG: immediate-early gene
ICAM-1: intercellular adhesion molecule-1
IFN-c: interferon-c
IL-1β: IL-6, IL-10, IL-17, interleukins
MC2R: melanocortin 2 receptor
MACS: mild autonomous cortisol secretion
NR3C2: mineralocorticoid superfamily
MDSCs: myeloid-derived suppressor cells
NK: natural killer cells
PVN: paraventricular nucleus
PI3K: phosphoinositide 3-kinase
pGR: phospho-Ser145-GR
PAR: poly(ADP-ribose
PARP: poly(ADP-ribose) polymerase
PR: progesterone receptor
POMC: proopiomelanocortin
PKA: protein kinase A
RNS: reactive nitrogen species
ROS: reactive oxygen species
ROR-1: Receptor tyrosine kinase -like orphan receptor-1
Tregs: regulatory T cells
RFS: relapse-free survival
SGRM: selective GR modulator
SCC: squamous carcinoma cells
SGRMs: selective GR modifiers
StAR: steroid acute regulatory protein
CYP11B1: steroid C-11 hydroxylase
CYP17A1: steroid C-17 hydroxylase
CYP21A2: steroid C-21 hydroxylase
Ia: surface I-region-associated antigens
TJs: tight junction proteins
TFs: transcription factors
TNBC: triple negative breast cancer
TME: tumor microenvironment
TNF: tumor necrosis factor
TNFα: tumor necrosis factor-α
TALs: tumor-associated lymphocytes
TILs: tumor-infiltrating lymphocytes
UCN1: UCN2, UCN3, urocortins 1, 2, and 3
VitD: vitamin D
VDR: vitamin D receptor
